# Genetic profile of progressive myoclonic epilepsy in Mali reveals novel findings

**DOI:** 10.3389/fneur.2024.1455467

**Published:** 2024-09-25

**Authors:** Lassana Cissé, Salia Bamba, Seybou H. Diallo, Weizhen Ji, Mohamed Emile Dembélé, Abdoulaye Yalcouyé, Toumany Coulibaly, Ibrahima Traoré, Lauren Jeffries, Salimata Diarra, Alassane Dit Baneye Maiga, Salimata Diallo, Karamoko Nimaga, Amadou Touré, Oumou Traoré, Mahamadou Kotioumbé, Emily Kathryn Mis, Cheick Abdel Kader Cissé, Cheick Oumar Guinto, Kenneth H. Fischbeck, Mustafa K. Khokha, Saquib A. Lakhani, Guida Landouré

**Affiliations:** ^1^Faculté de Médecine et d’Odontostomatologie, Université des Sciences, des Techniques et des Technologies de Bamako (USTTB), Bamako, Mali; ^2^Service de Médecine, Hôpital Nianankoro Fomba de Ségou, Ségou, Mali; ^3^Department of Pediatrics, Pediatric Genomics Discovery Program (PGDP), Yale University School of Medicine, New Haven, CT, United States; ^4^Service de Neurologie, Centre Hospitalier Universitaire Gabriel Touré, Bamako, Mali; ^5^Department of Genetic Medicine, McKusick-Nathans Institute, Johns Hopkins University School of Medicine, Baltimore, MD, United States; ^6^Service de Neurologie, Centre Hospitalier Universitaire Point G, Bamako, Mali; ^7^Clinique médicale Dinandougou, Marka Coungo, Mali; ^8^Service de Pédiatrie, Centre Hospitalier Universitaire Gabriel Touré, Bamako, Mali; ^9^Neurogenetics Branch, NINDS, NIH, Bethesda, MD, United States

**Keywords:** progressive myoclonic epilepsy, genetic, novel variants, Mali, West Africa

## Abstract

**Background and objectives:**

Progressive myoclonic epilepsy (PME) is a group of neurological disorders characterized by recurrent myoclonic seizures with progressive neurological deterioration. We investigated the genetics of three unrelated patients with PME from Mali, a country in sub-Saharan Africa highly underrepresented in genetic and genomic research.

**Methods:**

Participants were carefully examined and phenotyped. DNA was obtained for genetic analysis including whole exome sequencing (WES). *In silico* prediction tools and ACMG criteria were used to assess the deleteriousness of putative candidate variants.

**Results:**

Pedigree analysis suggests autosomal recessive inheritance patterns for one family and sporadic forms of PME for the two other cases. WES identified novel homozygous missense variants in all the three patients, one each for *NHLRC1*, *EPM2A*, and *NEU1*. The sequence variants segregated with PME in each family and *in silico* studies including protein 3D structures, CADD scores and ACMG criteria suggested that they were damaging.

**Discussion:**

PME is a group of clinically heterogeneous neurological disorders. Most reported cases in the literature are from European background with only a few cases described in North Africa. We report here novel pathogenic variants in three different genes causing PME phenotypes in three unrelated Malian patients, suggesting that genetic studies of underrepresented populations may expand the genetic epidemiology of PME. These findings also emphasize the need for inclusive genetic research to ensure a more targeted diagnostic and therapeutic approaches for diverse patient populations.

## 1 Introduction

Progressive myoclonic epilepsy (PME) is a group of clinically and genetically heterogeneous disorders characterized by recurrent myoclonic seizures, and progressive neurological and cognitive deterioration. PMEs are uncommon disorders and the diagnosis is challenging since symptoms are often non-specific and can mimic other neurological conditions (1). Although the etiology is undetermined in many patients, the gene defects for several PMEs (Unverricht-Lundborg disease, Lafora disease, several forms of neuronal ceroid lipofuscinoses, myoclonic epilepsy with ragged-red fibers [MERRF], and type 1 sialidoses) have been identified, leading to improvement in diagnosis and therapy (2, 3).

Despite this progress, genetically confirmed cases of PME remain scarce in several sub-Saharan Africa (SSA) countries, including Mali, highlighting a significant gap in the exploration of molecular neurological disorders in the region. The limited availability of specialists and genetic diagnostic tools contributes to this knowledge deficit (4). Nevertheless, Mali’s diverse population, characterized by at least 14 distinct ethnic groups, exhibits homogeneous cluster populations with specific phenotypic traits and an increased prevalence of recessive disorders in certain areas (4). This presents a unique opportunity to identify unique PME-associated genotypes. In this study, we present novel findings from three Malian families with PME attributable to rare genetic variants.

## 2 Methods

### 2.1 Standard protocol approvals, registrations, and patient consents

This study was done in full compliance with the declaration of Helsinki and approval was obtained from the Ethics Committee of the Faculté de Médecine et d’Odontostomatologie, Université des Sciences, des Techniques et des Technologies de Bamako, Mali. Written informed consent/assent was obtained from all participants with agreement to share data.

### 2.2 Clinical and laboratory assessment

Patients were examined by neurologists, pediatricians, and medical geneticists. Blood chemistries, computed tomography (CT) scan, magnetic resonance imaging (MRI) and electroencephalography (EEG) were performed in selected available patients to refine the diagnosis or to rule out acquired causes of seizures.

### 2.3 Genetic analysis

**Figure 1:**
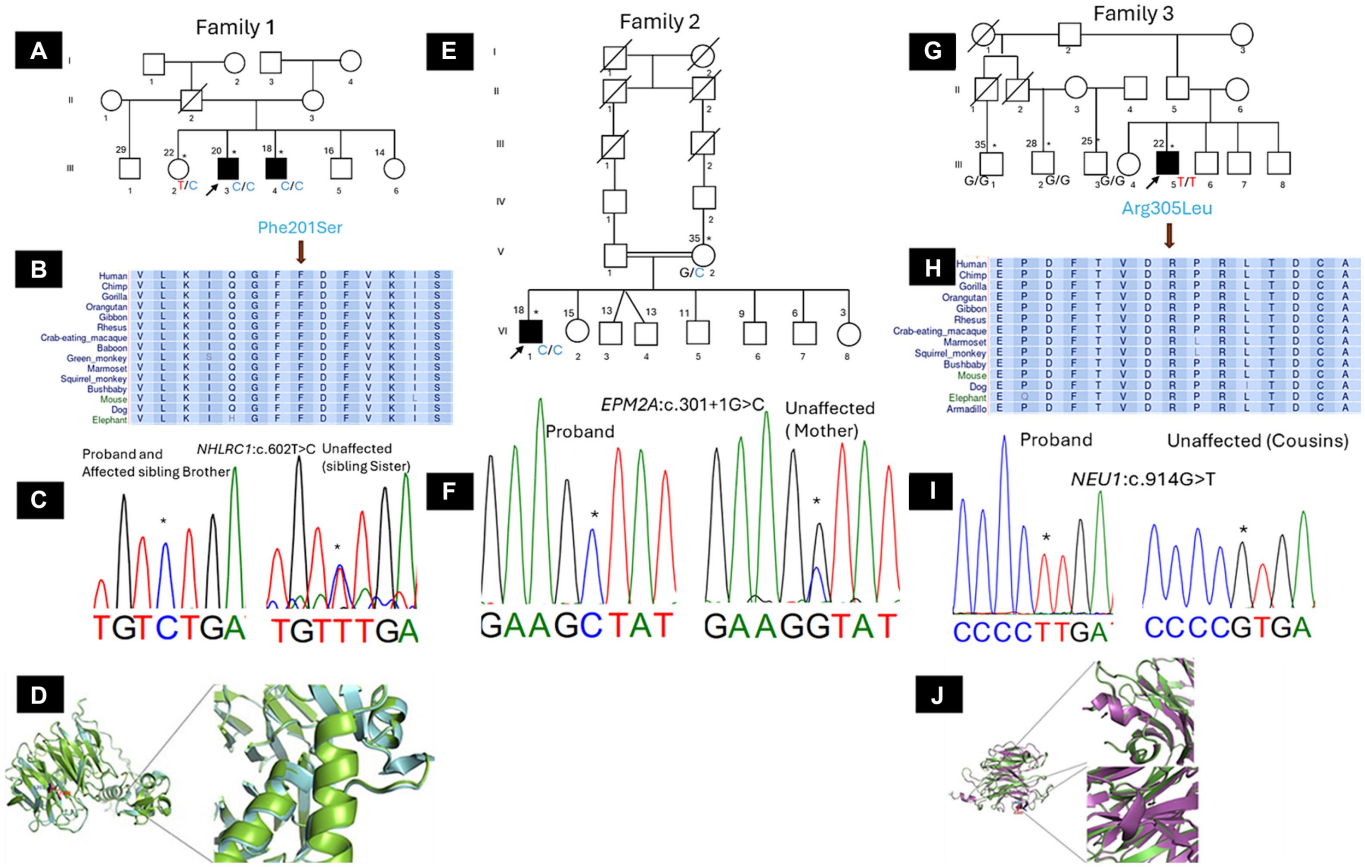
Phenotypic characteristics and genetic findings in families with PME (Family 1). **(A)** Pedigree of the family with Lafora disease with no reported consanguinity but two siblings having the disease (the black arrow indicates the proband and asterisks the participants seen in clinic). **(B)** The amino acid (Phe201) encoded at the variant site was highly conserved among mammals (Using the UCSC Genome Browser). **(C)** A chromatogram showing the changes, a homozygous C > T change (Phe201Ser). **(D)** Analyses of the secondary structures (ss) on 3D models revealed several major changes in the mutant (red) side chains when compared to the wild-type (blue) side chains of NHLRC1. Phenotypic characteristics and genetic findings in families with PME (Family 2). **(E)** Pedigree of the family with Lafora disease with an affected member from parental consanguinity (the black arrow indicates the proband and asterisks the participants seen in clinic). **(F)** A chromatogram showing the changes, a homozygous splice site variant in *EPM2A* (*c.301 + 1G > C*). Phenotypic characteristics and genetic findings in families with PME (Family 3). **(G)** Pedigree of the family with Sialidosis type 1 with an affected member from a non-consanguineous marriage (the black arrow indicates the proband and asterisks the participants seen in clinic). **(H)** The amino acid (Arg305) encoded at the variant site was highly conserved among mammals (Using the UCSC Genome Browser). **(I)** A chromatogram showing the changes, a homozygous G > T change (Arg305Leu). **(J)** Analyses of the secondary structures (ss) on 3D models revealed several major changes in the mutant (red) side chains when compared to the wild-type (blue) side chains of NEU1.

DNA was extracted from peripheral blood using the QIAGEN Puregene Blood DNA kit C (Qiagen, Germantown, MD, USA) following the manufacturer’s instruction. WES was performed in all affected individuals and the available family members (Figure 1A: III.2, III.3, III.4; Figure 1B: V.2, VI.1; Figure 1C: III.3, III.5). Sanger sequencing was performed to confirm the sequence variation and to check for segregation within the families (Family 1: III.2, III.3, III.4; Family 2: V.2, VI.1; Family 3: III.1, III.2, III.3, III.5). Variant calling, annotation, and prioritization as well as prediction for deleteriousness are detailed in Supplementary material 1 (5, 6). Variants were selected based on the suspected inheritance pattern within the family and the number of affected individuals, considering *de novo,* hemizygous, homozygous, and compound heterozygous variants, according to the following criteria: (i) missense, nonsense, frameshift, non-frameshift, or splicing site variants, (ii) SNPs with a minor allele frequency of <0.0005 in the SNP database were selected and filtered, (iii) In order to assess deleterious effects of variants, bioinformatics tools were applied including combined annotation dependent depletion [CADD pathogenicity prediction scoring >20 predicts the top 1% of deleterious variants^1^ (7)], sorting intolerant from tolerant,^2^ MutationTaster,^3^ protein variation effect analyzer,^4^ polymorphism phenotyping v2.^5^ The final candidate variant was evaluated according to ACMG (American College of Medical Genetics and Genomics) variant interpretation guidelines (8).

For genes with predicted nonsynonymous protein coding variants (*NHLRC1* and *NEU1*), secondary protein structures were predicted on PSIPRED Workbench.^6^ Protein three-dimensional (3D) structures were modeled on Swiss-model server and visualized using Pymol software (9). More details are provided in Supplementary material 1.

## 3 Cases description

Phenotypic and genetic features are summarized in Table 1.

**Table 1:** Phenotypic and genetic findings in patients with PME.

Patients	Clinical examination findings											Laboratory findings		
	Age (y)	Sex	Age of onset (y)	First symptom	Tremor	Myoclonic jerks	Dysarthria	Ataxia	Nystagmus	GTCS	Cognitive decline	EEG	Brain CT scan	Variant
F1: III.3	20	M	15	GTCS	None	Yes	Yes	Yes	No	Yes	Yes	Generalized spike waves	Normal	NHLRC1 (c.T602C; p.Phe201Ser) Homozygous
F1: III.4	18	M	16	GTCS	None	Yes	Yes	Yes	No	Yes	Yes	Generalized spike waves	Normal	
F2:VI.1	18	M	14	GTCS and tremor	Yes	Yes	Yes	Yes	Yes	Yes	Yes	Slow background activity with generalized spikes waves	Cerebellar atrophy	EPM2A (c.301 + 1G > C) Homozygous
F3: III.5	22	M	15	Tremor	Yes	Yes	Yes	Yes	Yes	Yes	None	Slow background with generalized spike waves	Normal	NEU1 (c.914G > T; p.Arg305Leu) Homozygous

F, family; y, year; GTCS, generalized tonic clonic seizures; CT, Computed tomography.

### 3.1 Case 1 (Family 1)

This family had two male affected siblings aged 18 and 20 years from Dogon ethnicity who were referred to our clinic for genetic exploration of pharmaco-resistant generalized tonic clonic seizures which started at the ages of 15 and 16 years, respectively (Figure 1A). No parental consanguinity was reported but parents are from the same ethnic background and the same geographical area. Neurological examination found generalized myoclonic jerks with neuropsychiatric symptoms including aggressiveness, slurred speech and cognitive decline. EEG revealed a normal background with generalized spike waves, suggesting generalized epilepsy. Brain CT scan was normal. Despite antiseizure medications including sodium valproate and clonazepam, their neurological condition worsened progressively and died at ages 21 and 23, respectively. WES was performed on both patients to identify the genetic cause of PME. Shared variants between the two patients were selected from sequencing results through rigorous bioinformatics analysis. *In silico* prediction revealed five deleterious variants. According to ACMG classification, three variants were selected: one likely pathogenic and two variants of uncertain significance (VUS) (Supplementary Table S1). The likely pathogenic variant, classified by ACMG criteria (PM1, PM2, PP1, PP3, PP4), was identified in *NHLRC1*, c.602 T > C (p.Phe201Ser) in both affected siblings, segregating with the disease in the family. The amino acid (Phe201) which was highly conserved among mammals using the UCSC Genome Browser and the changes, a homozygous C>T change (Phe201Ser) are shown in the Figure 1B,C. Substituting a non-polar Phenylalanine with a polar Serine was predicted to affect protein structure (Figure 1D; Supplementary Figure S1).

### 3.2 Case 2 (Family 2)

The proband was an 18-year-old male from Soninké ethnicity with myoclonic epilepsy that began at 14 years of age with initial symptoms of mild hand tremors causing objects like pens and bowls to slip (Figure 1E). A chromatogram showing the changes, a homozygous splice site variant in EPM2A (c.301+1G>C) is depicted in Figure 1F. He is from an eventful pregnancy and delivery with consanguineous parents. Clinical examination found intellectual disability, dysarthria, and visual impairment. While muscle strength was preserved, brisk reflexes were noted in the upper and lower limbs, in addition to tremors and clonus in the latter. He had difficulties standing, and was unable to walk. EEG showed a slow background activity with generalized spikes waves (Supplementary Figure S2) and brain imaging showed cerebellar atrophy. Treatment was initiated with sodium valproate, clonazepam and piracetam but myoclonus and generalized tonic clonic seizures recured frequently and the patient consequently died at age 20. WES was performed on the proband and his unaffected mother. Shared monoallelic variants were excluded from the sequencing results through rigorous bioinformatics analysis. *In silico* prediction revealed 24 deleterious variants. According to ACMG classification, nine variants were selected: one likely pathogenic and eight variants of uncertain significance (VUS) (Supplementary Table S2). The likely pathogenic variant, classified by ACMG criteria (PVS1, PM2, PP4), was identified in *EPM2A*, c.301 + 1G > C, with a SpliceAI score of 0.96 for donor loss and 0.2 for donor gain.

### 3.3 Case 3 (Family 3)

The proband was a 22-year-old male with a 7-year history of dysarthria and myoclonic jerks in the peribuccal area (Figure 1G). He was from Bambara ethnicity. There was no parental consanguinity and no familial history of the disease. The patient developed generalized myoclonic jerks that were exacerbated by strong emotions and sensory stimulations, as well as generalized tonic clonic seizures with generalized rigidity and hypertonia leading to severe disability. Reflexes and coordination were difficult to evaluate due to exacerbated myoclonic jerks on attempt. EEG showed slow background with generalized spike waves and brain CT scan was normal. Treatment with sodium valproate and clonazepam led to an initial improvement of myoclonic jerks and seizures. However, his neurological condition worsened gradually and he died 5 years later. WES was performed on the proband and one of his unaffected paternal cousins. Shared monoallelic variants were excluded through rigorous bioinformatics analysis. *In silico* prediction revealed 28 deleterious variants. According to ACMG classification, 23 variants were selected: one likely pathogenic and 22 variants of uncertain significance (VUS) (Supplementary Table S3). The likely pathogenic variant, classified by ACMG criteria (PM1, PM2, PM5), was identified in *NEU1*, c.914G > T (p.Arg305Leu). The amino acid (Arg305) conservation among mammals and a chromatogram showing the changes, a homozygous G>T change (Arg305Leu) are depicted in Figure 1H,I. The predicted 3D structure of variant NEU1 showed significant changes, gaining new helical structures from switching positively charged Arg305 to the non-polar Leu305 (Figure 1J; Supplementary Figure S3).

Variant’s pathogenicity criteria is summarized in Supplementary Table S4.

## 4 Discussion

Progressive Myoclonic epilepsies are a group of clinically and genetically heterogeneous disorders characterized by myoclonus, seizures, and progressive neurological decline with variable onset in childhood or adolescence. Molecularly, several common and rare PMEs including Unverricht-Lundborg disease (ULD), Lafora disease (LD), several forms of neuronal ceroid lipofuscinoses (NCLs), myoclonic epilepsy with ragged-red fibers [MERRF], type 1 sialidose (ST-1), North Sea PME, Acid ceramidase deficiency (Farber disease/SMA-PME) have been characterized with a wide phenotype variability (10). They are mostly transmitted following an autosomal recessive manner with rare cases of mitochondrial and autosomal dominant inheritance (10). Despite significant progress being made to understand the landscape of molecular defects underlying PME, only a few cases have been described in Africa (11, 12). To the best of our knowledge, this is the first report of PME caused by variants in *NEU1* and *EPM2A* on the African continent and the second reported PME caused by variant in *NHLRC1* in the Malian population (11–13).

Variants in *NHLRC1* and *EPM2A* are known to cause Lafora disease through synaptic vesicle formation and neurotransmitter release. The *NHLRC1* gene encodes malin, an E3-ubiquitin ligase and the *EPM2A* gene encodes laforin, a dual-specificity protein phosphatase. Mutation of these two genes results in the accumulation of polyglucan bodies called Lafora bodies within neurons and that can cause a range of neurological symptoms (14). Here, we report the second mutation in *NHLRC1* and the first mutation in *EPM2A* in the Malian population. Notably, both the previously reported amino acid change (p.His187Pro) and the (p.Phe201Ser) variant we report here are localized in a highly conserved NHL domain (12). Like the previously reported *NHLRC1* variant in Mali, the case we present here showed a severe phenotype with a shorter disease duration while mild disease phenotype have been associated to *NHLRC1* variants in other continents (10, 12).

*NEU1* is known to encode for a sialidase enzyme that is involved in the breakdown of sialic acid, a sugar found on the surface of cells. Damaging variants in this gene lead to the accumulation of sialic acid in the brain and cause a form of neurodegenerative disease known as sialidosis and characterized by cognitive impairment, seizures, and motor problems. Mutations in this gene were previously associated with neurodegenerative diseases including Gaucher disease and Alzheimer’s disease (15). This is the first mutation in *NEU1* reported in the Malian population. The disease duration was 12 years in our patient, however more prolonged durations up to 30 years have been reported in other populations (16). This could be explained by genetic or environmental modifiers. In addition, difficulties in accessing specialized healthcare services could also play a role in our context.

Overall, the discovery of these variants in PME patients from different ethnic backgrounds (Bambara, Dogon, and Soninké) for the first time in Mali or in Africa further supports a role for the identification of genetic variants in African PME. The increasing access to sequencing technologies may unravel genetic variants with significant implications for the understanding and treatment of PME, and will trigger hope for the inclusion of African patients in the future therapeutic developments for this debilitating disease.

## Data Availability

The original contributions presented in the study are publicly available. This data can be found here: https://www.ncbi.nlm.nih.gov/clinvar/ with accession numbers SCV005200387.1, SCV005200391.1 and SCV005200399.1 respectively for variants in NHLRC1 (Case 1), EPM2A (Case 2) and NEU1 (Case 3).

## Footnotes

^1^https://cadd.gs.washington.edu/home.

^2^SIFT; https://sift.bii.a-star.edu.sg/.

^3^http://www.mutationtaster.org/.

^4^PROVEAN; http://provean.jcvi.org/index.php.

^5^PolyPhen-2; http://genetics.bwh.harvard.edu/pph2/.

^6^http://bioinf.cs.ucl.ac.uk/psipred/.

## Supplementary material

Supplementary Figure S1 The predicted 3D structure of variant NHLRC1. The Substituting a non-polar Phenylalanine with a polar Serine was predicted to affect protein structure apparent in the refined 3D structure **(A)**, which is predicted to impact the overall folding of the protein **(B, C)**. In addition, the change of a Non-polar and hydrophobic amino acid (aa) Phenylalanine into a polar aa Serine may impact the binding interaction of the protein.

Supplementary Figure S3 The 3D structure of the mutant NEU1 revealed additional major changes including a gain of new helical structures **(A)**. Although both wildtype aa R305 and mutant L305 are not involved directly in bonding interaction **(B,C)**, the change of a positively charged aa (Arginine) to a non-Polar and hydrophobic aa (Leucine) is predicted to impact the protein interaction ability.
