# Supplementary figures and images for: Genetic profile of progressive myoclonic epilepsy in Mali reveals novel findings
Source: Front Neurol. 2024 Sep 25;15:1455467. doi: 10.3389/fneur.2024.1455467 (PMC11461190; doi:10.3389/fneur.2024.1455467)

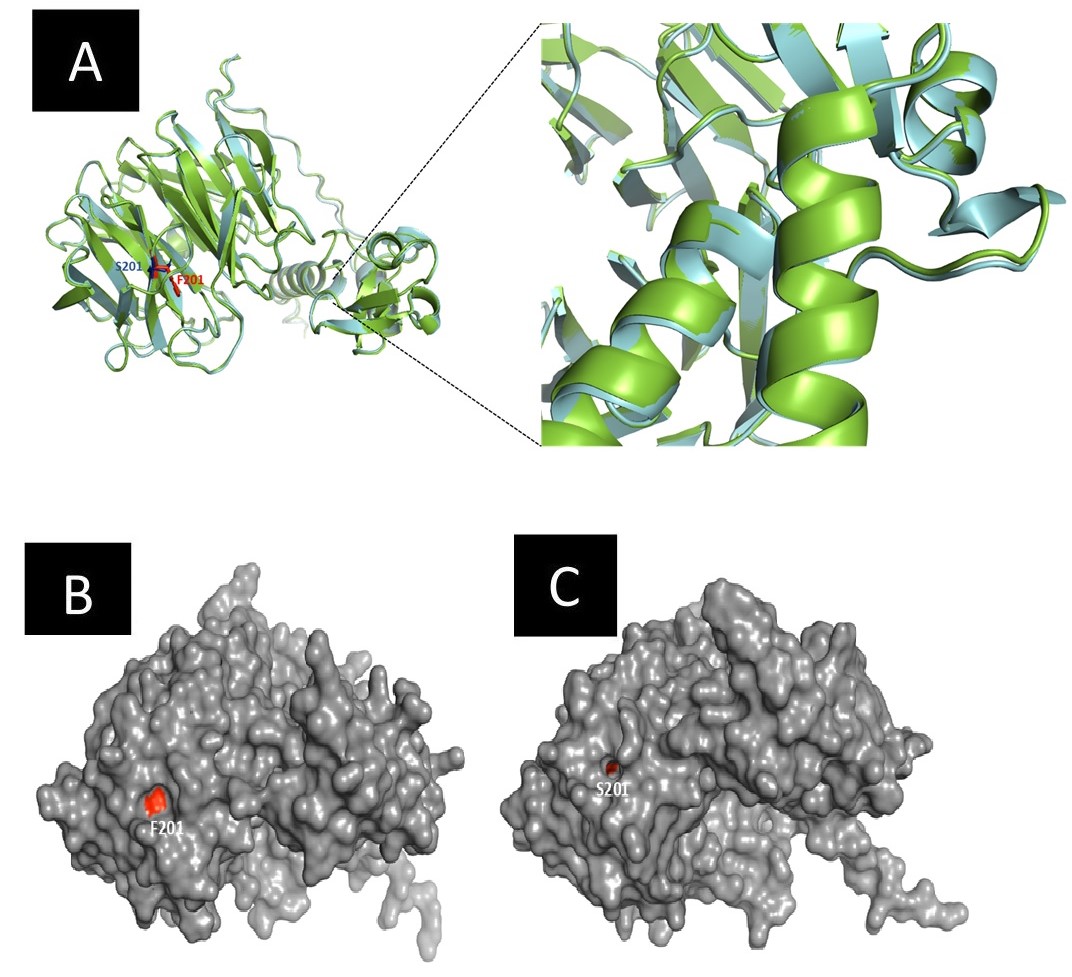

Supplement: Supplementary Figure S1 — The predicted 3D structure of variant NHLRC1. The Substituting a non-polar Phenylalanine with a polar Serine was predicted to affect protein structure apparent in the refined 3D structure (A), which is predicted to impact the overall folding of the protein (B, C). In addition, the change of a Non-polar and hydrophobic amino acid (aa) Phenylalanine into a polar aa Serine may impact the binding interaction of the protein. [file Image_1.jpg]

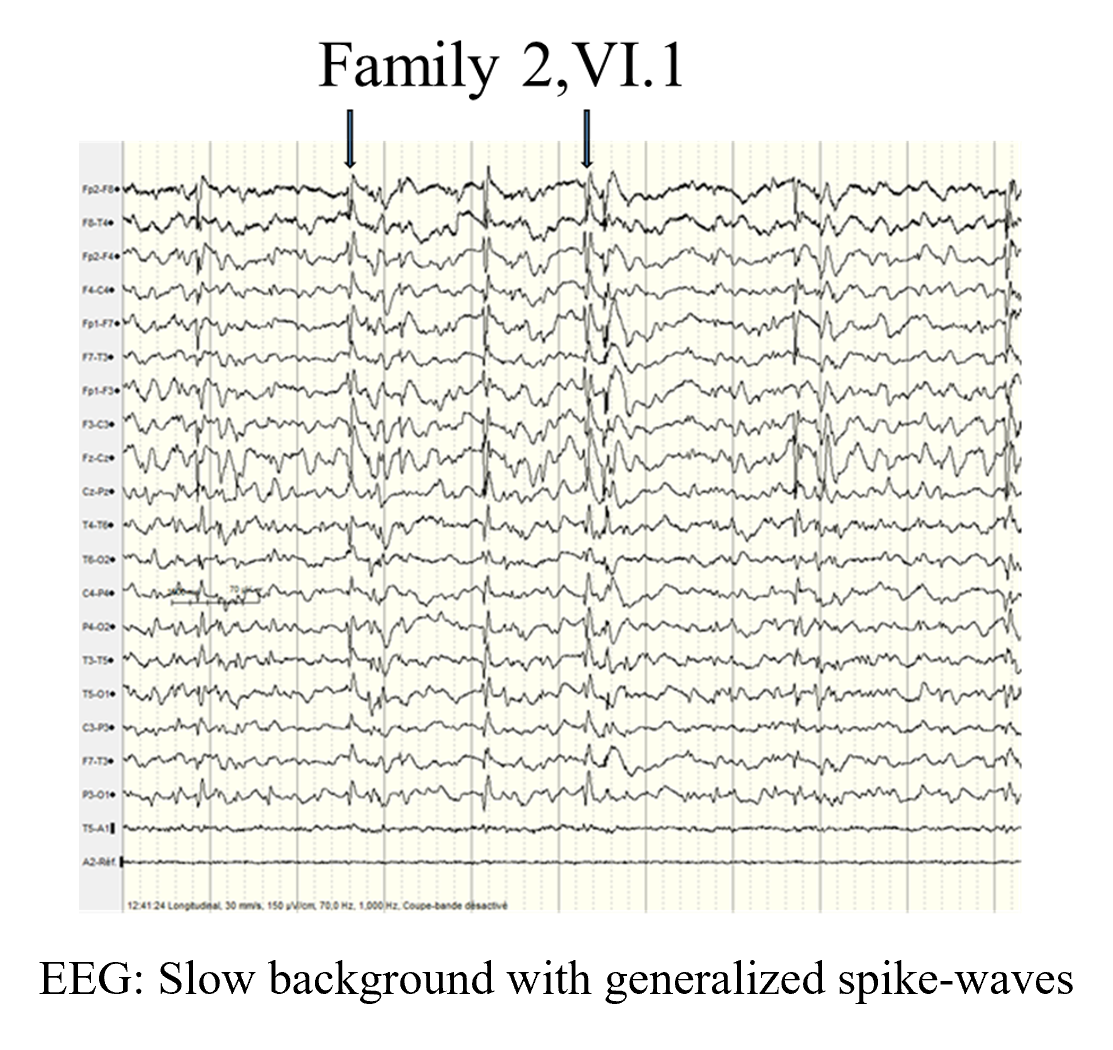

Supplement: Supplementary Figure S3 — The 3D structure of the mutant NEU1 revealed additional major changes including a gain of new helical structures (A). Although both wildtype aa R305 and mutant L305 are not involved directly in bonding interaction (B,C), the change of a positively charged aa (Arginine) to a non-Polar and hydrophobic aa (Leucine) is predicted to impact the protein interaction ability. [file Image_2.tif]

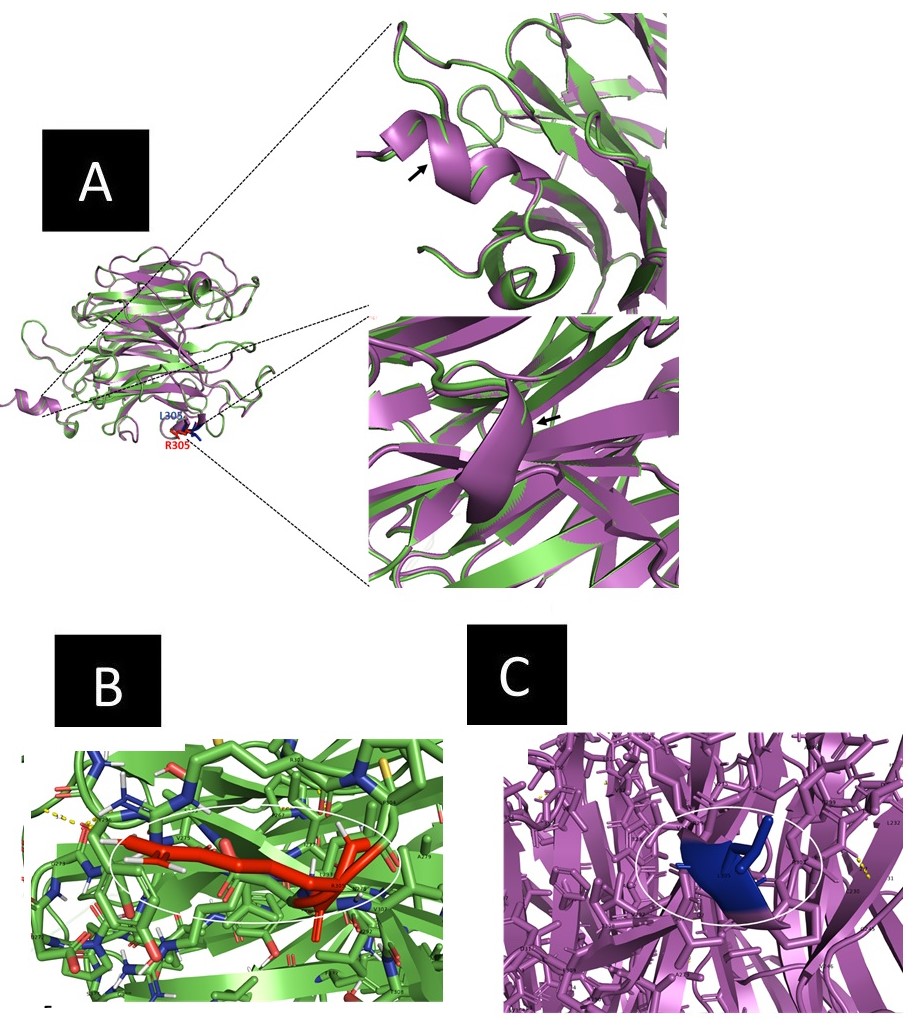

Supplement: Supplementary file 3 [file Image_3.jpg]
