# Supplementary material for: Genetic profile of progressive myoclonic epilepsy in Mali reveals novel findings
Source: Front Neurol. 2024 Sep 25;15:1455467. doi: 10.3389/fneur.2024.1455467 (PMC11461190; doi:10.3389/fneur.2024.1455467)
Supplement: Supplementary file 7 [file Table_4.docx]

**Supplementary Table S4**

| **Prediction tools** | Family 1  *NHLRC1*  c.T602T>C; p.Phe201Ser) | Family 2  *EPM2A*  c.301+1G>C | Family 3  *NEU1*  c.914G>T; p.Arg305Leu) |
| --- | --- | --- | --- |
|  | Homozygous | Homozygous | Homozygous |
| CADD | 29.5 | 24.8 | 24.6 |
| PolyPhen2 | Damaging | - | Damaging |
| BayesDel addAF | Damaging | Pathogenic | Damaging |
| DANN | Damaging | Damaging | Damaging |
| DEOGEN2 | Damaging | Damaging | Damaging |
| FATHMM | Damaging | Damaging | Damaging |
| M-CAP | Damaging | - | Damaging |
| MutationTaster | Disease causing | Damaging | Disease causing |
| PrimateAI | Damaging | - | Damaging |
| SIFT | Damaging | - | Damaging |
| REVEL | Pathogenic | - | - |
| ACMG Classification | Likely Pathogenic | Likely Pathogenic | Likely Pathogenic |
