# Supplementary material for: Genetic profile of progressive myoclonic epilepsy in Mali reveals novel findings
Source: Front Neurol. 2024 Sep 25;15:1455467. doi: 10.3389/fneur.2024.1455467 (PMC11461190; doi:10.3389/fneur.2024.1455467)
Supplement: Supplementary file 8 [file Data_Sheet_1.docx]

**Genetic analysis**

**Whole-Exome Sequencing**

WES was performed in all participants which have been seen in clinic (Figure 1A: III.2, III.3, III.4; Figure 1E: V.2, VI.1; Figure 1G: III.3, III.5). Exomes were captured using IDT xGen exome target capture kit, providing > 96% coverage of RefSeq coding bases with at least 20 independent reads. Enriched libraries were sequenced (2x100) on Illumina HiSeq 4000 instruments with an average of 6 gigabases per exome, giving 50x coverage at 90% of reads. The Varscan.v2.3.9.jar tools were used for variants calling**^7^** and annotation was done using ANNOVAR (2014-11-12) to include information regarding the gene, chromosomal coordinate(s), variants, type of mutation (frameshift, nonsense, nonsynonymous, splicing, and synonymous); and 16 predictions of the variant from multiple algorithms, allele frequencies in different databases including gnomAD, Exome Sequencing Project, dbSNP, 1000 Genomes, Complete Genomics, Exome Aggregation Consortium, and annotation of variants in clinical mutation database(ClinVar). Considering the variants annotated, we used the R program to prioritize candidate variants based on the inheritance pattern, annotation, population frequency, and gene expression information. We finally considered pathogenic variants on the gene that fit with the family disease classified according to the guidelines of the American College of Medical Genetics and Genomics (ACMG). The variants identified were then analyzed using bioinformatics tools to predict their potential impact on the protein structure and function such as Polyphen-2, and Combined Annotation-Dependent Depletion (CADD v1.6).

**Protein structure analysis**

Protein sequences NP_940988.2 of NHLRC1 and NP_000425.1 of NEU1 were retrieved from the National Center of Biotechnology Information (NCBI) in FASTA format. Secondary structures of wildtype and mutant proteins were predicted on PSIPRED Workbench (http://bioinf.cs.ucl.ac.uk/psipred/). The Three-dimensional (3D) structure of mutant proteins was modeled on the SWISS-MODEL server using AlphaFold structures Q6VVB1, Q99519, and P15328 as templates, respectively. Then, 3D structures were refined on the GalaxyWeb server (https://galaxy.seoklab.org/). PyMOL software was used for protein visualization, structural and hydrogen bonds analysis (Schrödinger. PyMOL | pymol.org. 2019).
